# Erythropoietin in the intensive care unit: beyond treatment of anemia

**DOI:** 10.1186/2110-5820-1-40

**Published:** 2011-09-23

**Authors:** Nimesh SA Patel, Massimo Collino, Muhammad M Yaqoob, Christoph Thiemermann

**Affiliations:** 1 Centre for Translational Medicine & Therapeutics, Queen Mary University of London, William Harvey Research Institute, Barts and The London, London, UK; 2 Department of Anatomy, Pharmacology and Forensic Medicine, University of Turin, Turin, Italy

## Abstract

Erythropoietin (EPO) is the major hormone stimulating the production and differentiation of red blood cells. EPO is used widely for treating anemia of critical illness or anemia induced by chemotherapy. EPO at pharmacological doses is used in this setting to raise hemoglobin levels (by preventing the apoptosis of erythroid progenitor cells) and is designed to reduce patient exposure to allogenic blood through transfusions. Stroke, heart failure, and acute kidney injury are a frequently encountered clinical problem. Unfortunately, in the intensive care unit advances in supportive interventions have done little to reduce the high mortality associated with these conditions. Tissue protection with EPO at high, nonpharmacological doses after injury has been found in the brain, heart, and kidney of several animal models. It is now well known that EPO has anti-apoptotic effects in cells other than erythroid progenitor cells, which is considered to be independent of EPOs erythropoietic activities. This review article summarizes what is known in preclinical models of critical illness and discusses why this does not correlate with randomized, controlled clinical trials.

## Introduction

Inflammation, renal failure, or blood loss due to frequent phlebotomies, gastric stress bleeding, coagulation disorders, or surgical procedures [1] often results in anemia in patients attending the intensive care unit (ICU). It is well established that the bone marrow of critically ill patients is able to respond to exogenously administered erythropoietin (EPO) to cause a significant increase in reticulocyte count and serum transferrin receptor concentration [2]. However, during the past two decades, our understanding of the actions of EPO has shifted from a belief that the hormone acts exclusively on erythroid progenitor cells to the knowledge that this agent exerts significant protection in conditions, such as sepsis, hemorrhagic shock, and ischemia/reperfusion injury (IRI) [3-9]. Many clinical settings are associated with IRI in the ICU. These include surgical procedures, transplantation, trauma-hemorrhage, and sepsis, among others. From a pathophysiologic perspective, ischemia and the associated tissue hypoxia leads to cell death, which are mediated by fundamental alterations in cellular homeostasis. Although no single factor has been identified as the critical mediator of cell death, depletion of cellular energy stores plays a major role [10]. Reestablishment of blood flow to an organ(s) after ischemia (reperfusion) is essential for the recovery and survival of the previously ischemic tissue. However, reperfusion itself often is harmful, because highly reactive molecules are produced that add injury to the previously ischemic organ (termed reperfusion injury). Furthermore, reperfusion can result in systemic alterations caused by the release/generation of vasoactive and/or proinflammatory mediators and cytokines (e.g., interleukin-6 [IL-6], tumor necrosis factor [TNF], high mobility group box protein-1 [HMGB-1]) within the previously ischemic tissue [11]. These events ultimately provoke a systemic inflammatory response syndrome (SIRS), which will invariably cause additional, remote organ injury (e.g., acute respiratory distress syndrome [ARDS]) [12]. The following review will highlight the experimental and clinical evidence for a tissue protective benefit of EPO, as opposed to its use for the treatment of anemia (see [13] for review), following various types of tissue injury.

### Experimental evidence

#### The brain

EPO and its receptor (EPOR) are weakly expressed in the human brain. However, their local production is rapidly induced in response to acute hypoxia, as evidenced by the increased expression of EPO in cerebrospinal fluid (CSF) or postmortem brain tissue in humans with stroke and hypoxia [14,15]. The first evidence that EPO is able to protect tissues/organs comes from several models of ischemic injury within the central nervous system (CNS). Cultured hippocampal and cerebral cortical neurons treated with EPO (100 pg/ml) were protected from glutamate neurotoxicity in a dose-dependent manner [16]. This protection was completely reversed by co-application of a soluble EPOR capable of binding and chelating EPO. Occlusion of the common carotid arteries in a gerbil induces a significant learning disability, which upon infusion of EPO into the lateral ventricles of these gerbils prevented the ischemia-induced learning disability and prevented the degeneration of hippocampal CA1 neurons [8]. Similarly, an infusion of soluble EPOR into animals that were only given a mild ischemic insult, which was insufficient to produce any damage by itself, caused CA1 neuronal degeneration. Using transgenic mice that constitutively express human EPO in the brain, without inducing excessive erythrocytosis, Kilic et al. [17] demonstrated that brain-specific overexpression of human EPO is associated with reductions in postischemic infarct volume and functional deficits and that the neuroprotective actions of EPO can be separated, at least in part, from its hematopoietic effects.

Despite previous evidence, it is now believed that EPO crosses the blood-brain barrier (BBB) in an injured brain. The first evidence for a neuroprotective effect of EPO by a peripheral route of administration was provided by Brines et al. (2000), who demonstrated in a focal stroke model a significant reduction in infarct volume by an intraperitoneally applied high dose of EPO (5000 IU/kg) up to 6 hours after reperfusion. Immunohistochemical detection of biotinylated EPO 5 hours after its intraperitoneal injection further provided evidence that EPO crosses the BBB [18]. EPO not only crosses the BBB but also can ameliorate BBB dysfunction evoked by IRI, as shown in a mouse model of acute ischemic stroke [19]. Besides, EPO has been reported to promote factors for angiogenesis, such as Tie-2 and angiopoietin-2, which may assist with the restoration of cerebral blood flow to preischemic levels [20].

These studies not only indicate that the survival of neurons after an ischemic insult is dependent on the formation of endogenous EPO but also indicate that exogenous EPO may protect the brain and, possibly, other tissues against IRI.

#### Cardiovascular system

In 2004, the expression of EPOR was thought to have been discovered in the rat heart [21] and a year later in the human heart [22], although expression of the EPOR in the heart remains inconclusive due to the poor specificity of the antibodies used for the immunostaining [23]. Before this knowledge, work on the heart was well underway and it had already been shown that EPO is able is reduce the increase in caspase activation and the subsequent apoptotic cell death caused by hypoxia and oxidative stress in rat ventricular myoblasts (H9C2 cells) [3,24]. The potential protective role of EPO in animal models of myocardial infarction has been assessed and beneficial effects have been observed regardless of whether EPO was administered before ischemia, at the onset of ischemia, or at reperfusion. A single bolus injection of EPO (5,000 IU/kg) 24 hours before the ischemic insult remarkably reduced infarct size and neutrophil infiltration [25]. We have reported that the administration of low doses of EPO (300 IU/kg i.v.) acutely upon reperfusion reduces the infarct size caused by coronary occlusion and reperfusion in the rat [3]. Similarly, chronic administration of EPO (5,000 IU/kg i.p.), daily for 7 days after myocardial ischemia, attenuates cardiomyocyte loss by approximately 50% [5]. There have been only two studies, thus far, comparing the protective effects of EPO when administered at different time points. Apoptosis was found to be further attenuated when EPO was administered after the start of reperfusion rather than just before ischemia [26]. Parsa et al. (2004) reported that the most significant protection was noted when EPO was given 12 hours before ischemia than when given at the onset of ischemia or reperfusion in rabbits [27]. Although there is clear evidence that EPO can prevent apoptosis in ventricular myoblasts and cardiomyocytes, as described above, endothelial cells express EPOR and hence, anti-apoptotic signaling can persist in the presence of EPO, which makes endothelial cells more resistant to ischemia-induced cell death [28]. Overall, these results suggest that EPO treatment is effective for both prevention and treatment of the ischemic episode.

The role of EPOR in cardioprotection has been investigated using transgenic animal models. Transgene-rescued EPOR null mutant mice when subjected to IRI showed a larger infarct size and reduced left ventricular (LV) function compared with wild-type mice with intact EPOR [29]. Tightly integrated with cardiac performance, pulmonary function also is enhanced during EPO administration, especially in the setting of IRI of the lung, thanks to EPO's ability to decrease TNF-α expression and inhibit leukocyte infiltration in lung tissue [30].

The therapeutic relevance of EPO in the clinical setting of cardiovascular diseases has been confirmed by studies in which EPO treatment was started 3 or 6 weeks after induction of myocardial infarction, when heart failure was fully established in mice [31,32]. In both reports, chronic EPO treatment significantly improved cardiac function and, interestingly, EPO's beneficial effects lasted for another 4 weeks even after EPO treatment was stopped. In 2008, Lipsic et al. demonstrated that a 3-weekly administration of low-dose EPO (equivalent to 80 U/kg) after myocardial infarction (with a permanent coronary artery ligation) did not raise hematocrit levels but was able to improve cardiac function significantly. Such a low-dose of EPO was demonstrated as being able to induce neovascularization of the infarcted tissue, and this could, in part, be responsible for the tissue protective properties of EPO on cardiac function [33]. However, this is not the first time that EPO has been shown to be able to stimulate the proliferation of a number of cells, including endothelial and tubular epithelial cells. Circulating bone marrow-derived endothelial progenitor cells (EPCs) promote vascular reparative processes. EPO has been shown to be a mediator for the mobilization and proliferation of EPCs in both ischemic and nonischemic tissue [34-36]. However, neovascularization only seems to occur in ischemic tissue [6,36]. For instance, patients with unstable coronary heart disease have significantly higher serum levels of EPO, which has been shown to be associated with an increased amount of functionally active EPCs [6]. We have demonstrated that preconditioning mice with EPO (1,000 U/kg/day for 3 days) in a protocol that has been shown to mobilize EPCs [6] was associated with a greater degree of renal protection in a model of renal IRI (30 minutes ischemia and 24 hours reperfusion) compared with the administration of a single dose of EPO (1,000 U/kg) at the time of reperfusion [6].

Lipsic et al. also administered a higher dose of EPO (equivalent to 8,000 IU/kg), which significantly increased hematocrit levels but with the added benefit of further improvements in cardiac function and neovascularization compared with the low-dose EPO group [33]. This could be the result of the following one of two (or both) reasons: 1) an increased oxygen delivery capacity to the injured tissue as a result of increased numbers of circulating erythrocytes; or 2) a dose-dependent effect of EPO's tissue protective properties. This particular study has demonstrated that EPO treatment improves cardiac function and induces neovascularization in postmyocardial infarction heart failure, even at doses that do not increase hematocrit. EPOs ability to improve cardiac function, independent of erythropoiesis and the mobilization of bone marrow-derived cells, has been recently confirmed by a study demonstrating that EPO prevented cardiomyocyte apoptosis and stimulated angiogenesis after myocardial infarction by direct upregulation of angiogenic factors, such as VEGF and angiopoietin-1 expression [37].

Although time-limited treatment with high-dose EPO may be beneficial and safe during acute ischemic injury, if prolonged therapy is required (for instance during heart failure), drug regimens using low-dose EPO (or EPO devoid of erythropoietic effects) may be more suitable to avoid the adverse effects of the treatment (see [38] for review).

#### Kidney

Although it has been recognized for many years that EPO is produced in the adult kidney, mainly by peritubular interstitial cells and tubular epithelial cells, it was not until 1999 that the EPOR was reported to be expressed in tubular epithelial cells [39]. When rats were subjected to 45 minutes of bilateral renal ischemia, there was significant renal dysfunction and damage to the tubular architecture. However, this process was prevented by administration of 3,000 IU/kg of EPO 24 hours before the ischemic insult, which was attributed to an attenuation of apoptosis [40]. In a similar study, Vesey et al. were able to demonstrate an attenuation of apoptosis in the proximal tubule, proximal convoluted tubule, and outer medullary thick ascending limb with the administration of 5,000 IU/kg of EPO 30 min before a 30-min ischemic insult [41]. This beneficial effect of EPO was secondary to inhibition of the activities of caspase-3, -8, and -9 in the proximal tubule [9], as well as inflammation induced by neutrophils [7]. The protection afforded by EPO against renal IRI also might be partly due to its antioxidant effects, because EPO administration before the onset of renal ischaemia has been shown to induce a significant decrease in lipid peroxidation and an increase in the activity of endogenous antioxidant defense mechanisms, such as SOD and GSH, in rat kidney tissue [42]. The beneficial effects of EPO on renal dysfunction and hemodynamics have been recently confirmed in large-animal models of ischemic acute kidney injury (monkeys, pigs, and dogs [43-46]).

An interesting and widely accepted phenomenon of EPO is its ability to protect tissues after the onset of injury. Just like the studies in the brain and heart, the kidney is no exception. The administration of 5,000 IU/kg of EPO as late as 6 hours after a 30-min ischemic insult protects the kidney from dysfunction and injury, with no significant difference in the protection seen when EPO is administered on reperfusion [47].

EPO also is protective against medication-induced renal dysfunction, facilitating the recovery from cisplatin-induced acute kidney injury [48] and attenuation of renal interstitial inflammation and fibrosis in chronic cyclosporine nephropathy [49].

### Mechanisms

The evidence in favor for a direct anti-inflammatory effect of EPO is continually increasing and thus reclassifying EPO as a multifunctional tissue-protective cytokine. Data indicate that EPO may be of benefit in certain disease models in which excessive inflammation plays a key role. For instance, EPO has been demonstrated not only to decrease proinflammatory cytokine production but also to increase the expression of the protective cytokine IL-10 after myocardial IRI in rats [25]. A recent study has shown that EPO administration within 2 hours of a lipopolysaccharide (LPS) insult prevented apoptosis, excessive nitric oxide production, peroxynitrite formation, and tissue hypoxia, but without any significant alterations in tissue neutrophilia, NF-κB activation, or the increased serum levels of proinflammatory chemokines and HMGB-1 [4]. Much of this data is consistent with previous findings, which have shown that EPO (10 IU/ml) prevents the expression of iNOS protein induced by interferon-γ and LPS in rat oligodendrocytes [50]. In a model of zymosan-induced nonseptic shock, EPO (1,000 IU/kg) attenuated the degree of systemic injury, and this effect was attributed to a reduced nitrotyrosine and poly (ADP-ribose) polymerase staining in the tissue of target organs and a reduction in the level of circulating cytokines (TNF-α and IL-1β) in the plasma [51]. We have reported that the administration of EPO (300 IU/kg) after severe hemorrhage (e.g., upon resuscitation) reduces the organ injury and dysfunction caused by hemorrhagic shock. This beneficial effect of EPO was associated with a reduced activation of apoptosis secondary to prevention of the activation of caspases -3, -8, and -9 [3]. EPO (1,000 IU/kg/day) also reduces the joint injury and chronic inflammation in a rat model of type II collagen-induced arthritis. It is likely that the anti-inflammatory effects of EPO in this model of chronic inflammation are--at least in part--secondary to prevention of tissue injury (both apoptosis and necrosis), which would result in the reduced formation of proinflammatory cytokines in the circulation [52]. Finally, both inflammatory and vascular endothelial cells have been shown to express EPOR [53,54]. Although EPO and cytokines do not seem to directly modulate EPOR levels in normal tissue, EPO can strongly stimulate the expression of its own pharmacological target at low oxygen tensions in endothelial cells [55]. These findings are further supportive of its important role in modulating the excessive inflammatory response associated with IRI.

Many of these observed effects of EPO are dependent on Janus tyrosine kinase 2-activation (mediated by the EPOR) and the nuclear translocation of NF-κB [56]. In inflammatory scenarios, EPO may reduce the inflammation associated with shock and ischemic injury through a reduction in apoptotic cell death [57] via up-regulation of the anti-apoptotic proteins Bcl-2 and Bcl-XL [58]. EPO also prevents the release of the proapoptotic proteins cytochrome *c *and caspase-3 from the mitochondrial membrane by up-regulating the phosphoinositide 3 kinase and protein kinase B (PI3K/Akt) pathway [59-61]. Thus, increasing or decreasing the cytoplasmatic concentration of these proteins, respectively, will result in the inhibition of apoptosis [62]. In addition, a fairly recent study showed that up-regulation of the PI3K/Akt pathway is associated with the enhanced expression of endothelial nitric oxide synthase (eNOS) in isolated mice cardiomyocytes, which resulted in increased levels of nitric oxide [63]. In a more recent study, eNOS has been shown to integrate with the β common receptor [64], a receptor that has in recent years been shown to play a crucial role in EPO-mediated nonhematopoietic effects [65]. Activation of the β common receptor with EPO initiates increased interaction between the β common receptor and eNOS, resulting in the increased formation of nitric oxide. Nitric oxide not only directly limits the formation of free radicals but also promotes vasodilatation and could therefore limit hypoxic damage during vaso-occlusion [66]. In a transgenic mouse model overexpressing human EPO, the authors demonstrated that these mice fail to develop hypertension, stroke, myocardial infarction, or thromboembolism [67] but do have hematocrit levels of approximately 80% with generalized vasodilatation [68]. The inhibition of eNOS in these mice causes 100% mortality within 2 days and hence the generalized vasodilatation was attributed to an up-regulation of eNOS expression, increased NO production, and NO-mediated vascular relaxation compared with wild-type animals. Similarly, the administration of EPO to wild-type and eNOS^-/- ^mice before ischemia resulted in increased expression of eNOS and decreased infarct size only in the wild-type mice [63].

Although several experimental data suggest that EPO can induce cytoprotection through EPOR and/or the β common receptor as described above, there are still further investigations required to confirm the identity of EPO receptor subtypes as well as the different factors involved in the beneficial effects of EPO in several experimental models of IRI and inflammation.

### Clinical evidence

Considering all of the above-mentioned preclinical data, EPO is an attractive molecule for evaluation in several human disease conditions. The first of these conditions to evaluate EPO as a potential therapy was human stroke [69]. The purpose of the Göttingen EPO Stroke Study (a pilot noncontrolled study) was to determine whether EPO would be safe due to the possibility of raised hematocrit and subsequently increasing the likelihood of further transient ischemic attacks during a 30-day follow-up period. During the 30-day follow-up period hematocrit, hemoglobin, and red blood cell counts all remained normal despite a high infusion of EPO (100,000 IU over 3 days). This trial demonstrated that EPO is well tolerated and improves clinical outcome at 1 month as determined by neurological scoring and MRI. However, the more recent German Multicenter EPO Stroke Trial, which was designed to reproduce the results of the Göttingen EPO Stroke Study, has unexpectedly documented that a combination of EPO and recombinant tissue plasminogen activator (tPA) is not advantageous and may even be detrimental [70]. Patients were infused 40,000 IU of EPO over 30 min within the first 6 hours after the onset of symptoms and EPO (at a same dose) was readministered 24 and 48 hours later. During the 90-day follow-up period, the EPO arm demonstrated an increased risk of death, intracerebral hemorrhage, brain edema, and thromboembolic events. Moreover, patients who received both EPO and tPA (60% of patients) were significantly more likely to die than those receiving EPO only. It is possible that the beneficial effects of EPO were antagonized by tPA resulting in the higher mortality. Thus, caution is necessary when clinical trials with EPO in patients with ischemic stroke are conducted, and one must be vigilant for known and unknown side effects of this potential life-saving strategy.

With trauma being the leading cause of mortality and morbidity in the western world, it accounts for the highest incidence of deaths in Americans younger than age 34 years [71]. In critically ill trauma patients, anemia is very common and the CRIT study highlighted this very well, demonstrating that critically ill trauma patients were more likely to be transfused than critically ill nontrauma patients (55.4% vs. 44.1%) with a greater amount of blood transfusions (5.8 ± 5.5 units vs. 4.6 ± 4.9 units) [72]. This led to the hypothesis that treatment with pharmacological doses of EPO might decrease the exposure of patients to allogeneic blood and increase the hemoglobin level in anemic critically ill patients. This was essentially the beginning of three randomized studies (EPO-1, -2, and -3) involving 160, 1,302, and 1,460 patients, respectively. EPO-1 demonstrated a reduction in red blood cell transfusion that correlated with an increase in hemoglobin amongst critically ill patients treated with EPO [73]. These findings were confirmed in the second larger trial, EPO-2 [74]. Interestingly, this second trial demonstrated a survival benefit in critically ill trauma patients randomized to receive EPO treatment: at day 29 (deaths: placebo 8.9% vs. EPO 4.1%) and day 42 (deaths: placebo 10.4% vs. EPO 4.8%). However, the significance of this was not very clear, because the study did not collect specific information on trauma-specific variables that could have potentially affected patient outcome in the trauma cohort. In the third randomized study (EPO-3), admission groups were prospectively identified and randomization was stratified according trauma, medicine nontrauma and surgery nontrauma [75]. Patients were treated with EPO (40,000 IU/kg) once per week for 3 weeks. As with the EPO-2 study, a survival benefit was identified in critically ill trauma patients who received EPO: at day 29 (deaths: placebo 6.7% vs. EPO 3.5%), day 42 (deaths: placebo 7.2% vs. EPO 3.7%), and day 140 (deaths: placebo 9.2% vs. EPO 6.0%). However, on this occasion there was no reduction in red blood cell transfusion amongst patients treated with EPO despite an increase in hemoglobin concentration, which actually resulted in an increase in clinically relevant thrombovascular events (TVE: placebo 12.5% vs. EPO 16.4%). The data from EPO-3, therefore, suggests that the survival benefit was independent of any transfusion effects [76]. Moreover, this raises the issue of the tissue protective effects of EPO being overwhelmed by rising hematocrit if not controlled properly. Clearly, EPO mimetics that do not have the ability to raise hematocrit may be an ideal alternative in this area, such as the recently discovered pyroglutamate helix B surface peptide [77,78].

Randomized placebo-controlled trials with EPO in patients with myocardial infarction have been conducted in the Netherlands, Germany, and Japan [79-82]. Despite the apparent reduction of infarct size and improvement in LV function resulting from EPO treatment in animal models of myocardial infarction, overall these clinical trials did not demonstrate consistent results regarding LV function in the presence of EPO treatment. The two Japanese trials with low-dose EPO treatment, which were small-scale pilot studies, showed possible improvement of LV function, but the other two that used high-dose EPO treatment did not. In particular, the trial with the highest dose of EPO (approximately 100,000 IU) showed a tendency toward an increased incidence of adverse events, whereas the second study demonstrated that a single bolus with 60,000 IU i.v. of EPO was related to less major adverse cardiovascular events and a favorable clinical safety profile, although without any improvement in LV ejection fraction. Therefore, according to these recent clinical trials, the optimal dose of EPO remains to be determined. It can be speculated that measurement of LV function, such as LV ejection fraction, might not be the best surrogate marker to evaluate the possible beneficial effect of EPO during myocardial infarction. Large-scale clinical trials with a primary endpoint of cardiovascular events are needed to elucidate the efficacy of EPO administration as an adjunctive therapy of myocardial infarction. Recently, an open-label randomized 12-month trial (the Mechanisms of Erythropoietin Action in the Cardiorenal Syndrome [EPOCARES]) had been designed to discern hematopoietic from nonhematopoietic effects of EPO in patients with the combination of chronic heart failure and chronic kidney disease and mild anemia [83]. This study did not show significant influence of short- or long-term EPO therapy on reduced EPC levels in cardiorenal patients; however, this is not surprising because there are no preclinical data with EPO in a cardiorenal setting.

The EARLYARF trial was the first prospective, randomized, double-blind, placebo-controlled, parallel-group trial to study whether early treatment with EPO (500 IU/kg within 6 hours and again 24 hours later) could prevent the development of acute kidney injury in patients [84]. Although the trial found that EPO was not associated with an increase in clinically significant serious adverse events, unfortunately, the trial found no significant renoprotection or amelioration of acute kidney injury after intervention with EPO. At first glance this may seem quite disconcerting; however, on closer inspection it is possible to see that the EARLYARF trial had several methodological failings. The major limitation of the trial was the use of biomarkers (γ-glutamyltranspeptidase and alkaline phosphatase) that have been poorly validated, but at the inception of the trial were the only available biomarkers that were confirmed and rapidly measurable for renal injury [85]. It was only until after the trial was initiated that more relevant makers of renal injury were established (e.g., plasma neutrophil-gelatinase-associated lipocalin, and urinary kidney injury molecule-1, interleukin-18, and liver fatty acid-binding-protein) [86-90]. The use of such poorly understood biomarkers resulted in the inclusion of a more generalized critically ill population rather than those patients suspected of being in any real risk from acute kidney injury. The trial also included patients with reversible prerenal acute kidney injury based on biomarkers of injury alone. Despite there being any biomarker evidence of injury, a large majority of patients had preserved renal sodium reabsorption. This, subsequently, reduces the power of the study with respect to EPO efficacy. In addition, there were some baseline differences between placebo and EPO-treated cohorts. The placebo group was significantly younger than those treated with EPO, and a significant proportion of the EPO-treated group (23.8%) had sepsis. Finally, the first administration of EPO (at 6 hours) was at the later end of the current theoretical interval [47] during which an early benefit might be detected. All in all, these limitations amongst others demonstrate that using a single composite biomarker with a brief postinjury profile was insufficient for risk stratification in a population with a heterogeneous onset of injury [84]. A more extensive trial is required, with valid biomarkers/endpoints [91], to determine efficacy of EPO in acute kidney injury.

The most recent trial to be completed and analyzed this year was the Reduction of Infarct Expansion and Ventricular Remodeling with EPO After Large Myocardial Infarction (REVEAL) trial [92]. This trial included 222 patients with acute ST-segment elevation myocardial infarction who underwent successful percutaneous coronary intervention. EPO at a dose of 60,000 IU (~700 IU/kg) was administered within 4 hours of reperfusion. However, the primary outcome measure--infarct size--was unchanged between the placebo arm and EPO arm of the study during both the first week and at 12 weeks. In fact, the study revealed that older patients (> 70 years) receiving EPO had a 41.2% larger infarct size during the first week, suggesting that caution must be taken if EPO is administered to patients older than age 70 years, because may patients suffering from not only heart attacks but also stokes and acute kidney injury are generally within this age range. Interestingly, as seen in almost all clinical trials with EPO, the incidence of both adverse and serious adverse events was significantly higher in the EPO arm (94/125 patients) than the placebo arm (50/97 patients), with five deaths due to myocardial infarction, stroke, or stent thrombosis occurring in the EPO group only.

Alternatively, several large clinical trials of EPO performed to assess potential utility of normalizing the marginally low hemoglobin concentrations in patients with breast or head and neck cancers unexpectedly found an increase in mortality within the EPO arm due to tumor progression or significant thrombosis [93-95]. This was borne out in the TREAT study where the incidence of cancer deaths was higher in the EPO-treated group [96]. This is not surprising because preclinical data suggest that EPO is a very powerful promoter of tumor cell growth and is able to promote neovascularization to the site of the tumor, which can potentially increase metastasis.

It is interesting to consider why there are differences between animal and clinical studies. The most obvious assumption is that there are species differences, but the CRIT study has demonstrated a beneficial outcome with EPO despite the risk of adverse effects. The dose of EPO used in these studies also may play a major role. Animal studies use doses up to 5,000 IU/kg, and in clinical studies the dose rarely exceeds 1,000 IU/kg in any single administration. Timing of administration also may be very important; animal studies have taught us that there is a definitive window of treatment after injury.

## Conclusions

During the past decade, several key lines of evidence have strongly supported the view that endogenous EPO may play a crucial role in dampening the excessive tissue injury associated with by IRI and inflammation, but as yet there is no evidence that EPO may actually accelerate recovery after injury. Systemic administration of EPO has been found to be active in a large number of animal models associated with IRI, including stroke, myocardial infarction, acute kidney injury, hemorrhagic shock, and SIRS. However, the use of EPO in several large clinical trials for the treatment of anemia in cancer patients was associated with increased adverse events. A surgical trauma trial that showed increased survival in the EPO arm also showed that this was at the expense of a 40% increase in clinically significant thrombosis. Thus, although administration of EPO has potentially valuable tissue protective effects, clinical trials have shown that the administration of EPO is accompanied by significant adverse effects or demonstrate poor utility due to inadequate study design. Notably, these complications seem to be more frequent with high doses of EPO, such as those used in the tissue protection proof-of-concept trials. There is still a need for properly designed clinical trials to assess the efficacy of EPO beyond anemia. We recommend that novel adjuvant therapies should be explored to promote the effectiveness of endogenously secreted EPO in critical care settings to restore EPOs sensitivity at the tissue level. This would limit the use of high doses of exogenous EPO that can overwhelm the tissue-protective properties of EPO with its adverse side-effects.

## Competing interests

The authors declare that they have no competing interests.

## Authors' contributions

NSAP wrote the first draft of the manuscript. MC contributed to the first draft. MMY revised and modified the first draft. CT reviewed and modified the final draft.
